# The psychology of experimental psychologists: Overcoming cognitive constraints to improve research: The 47th Sir Frederic Bartlett Lecture

**DOI:** 10.1177/1747021819886519

**Published:** 2019-11-14

**Authors:** Dorothy VM Bishop

**Affiliations:** Department of Experimental Psychology, University of Oxford, Oxford, UK

**Keywords:** Reproducibility, replication, statistics, schemata, confirmation bias, moral judgements, publication bias, citation bias, simulation, Registered Reports, solutions, incentives

## Abstract

Like many other areas of science, experimental psychology is affected by a “replication crisis” that is causing concern in many fields of research. Approaches to tackling this crisis include better training in statistical methods, greater transparency and openness, and changes to the incentives created by funding agencies, journals, and institutions. Here, I argue that if proposed solutions are to be effective, we also need to take into account human cognitive constraints that can distort all stages of the research process, including design and execution of experiments, analysis of data, and writing up findings for publication. I focus specifically on cognitive schemata in perception and memory, confirmation bias, systematic misunderstanding of statistics, and asymmetry in moral judgements of errors of commission and omission. Finally, I consider methods that may help mitigate the effect of cognitive constraints: better training, including use of simulations to overcome statistical misunderstanding; specific programmes directed at inoculating against cognitive biases; adoption of Registered Reports to encourage more critical reflection in planning studies; and using methods such as triangulation and “pre mortem” evaluation of study design to foster a culture of dialogue and criticism.


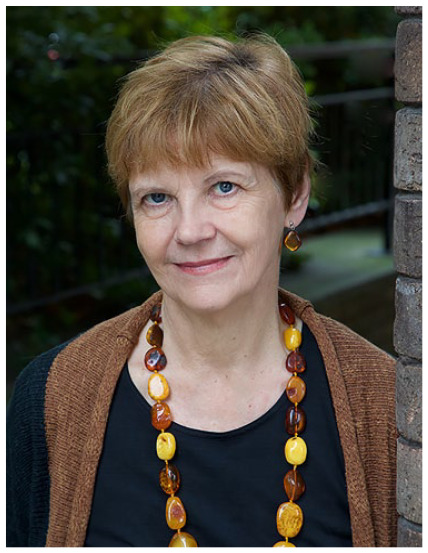


## Introduction

The past decade has been a bruising one for experimental psychology. The publication of a paper by Simmons, Nelson, and Simonsohn (2011) entitled “False-positive psychology” drew attention to problems with the way in which research was often conducted in our field, which meant that many results could not be trusted. Simmons et al. focused on “undisclosed flexibility in data collection and analysis,” which is now variously referred to as *p*-hacking, data dredging, noise mining, or asterisk hunting: exploring datasets with different selections of variables and different analyses to attain a *p*-value lower than .05 and, subsequently, reporting only the significant findings. Hard on the heels of their demonstration came a wealth of empirical evidence from the Open Science Collaboration (2015). This showed that less than half the results reported in reputable psychological journals could be replicated in a new experiment.

The points made by Simmons et al. (2011) were not new: indeed, they were anticipated in 1830 by Charles Babbage, who described “cooking” of data:

This is an art of various forms, the object of which is to give ordinary observations the appearance and character of those of the highest degree of accuracy. One of its numerous processes is to make multitudes of observations, and out of these to select only those which agree, or very nearly agree. If a hundred observations are made, the cook must be very unhappy if he cannot pick out fifteen or twenty which will do for serving up. (p. 178–179)

**Figure 1.**
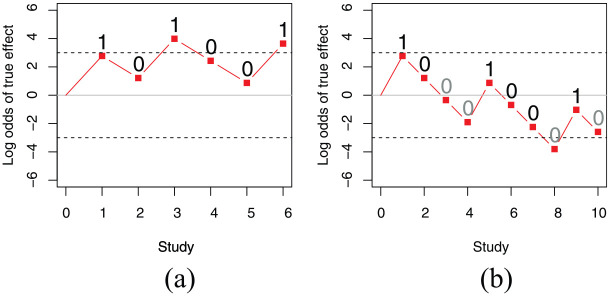
The impact of publication bias demonstrated with plots of cumulative log odds in favour of true versus null effect over a series of experiments. The log odds for each experiment can be computed with knowledge of alpha (.05) and power (.8); 1 denotes an experiment with significant difference between means, and 0, a null result. The starting point is zero, indicating that we assume a 50:50 chance of a true effect. For each significant result, the log odds of it coming from a true effect versus a null effect is log(.8/.05) = 2.77. For a null result, the log odds is log (.2/.95) = −1.55. The selected set of studies in panel (a) concludes with a log odds greater than 3, indicating that the likelihood of a true effect is 20 times greater than a null effect. However, panel (b), which includes additional null results (labelled in grey), leads to the opposite conclusion.

*P*-hacking refers to biased selection of data or analyses from within an experiment. Bias also affects which studies get published in the form of publication bias—the tendency for positive results to be overrepresented in the published literature. This is problematic because it gives an impression that findings are more consistent than is the case, which means that false theories can attain a state of “canonisation,” where they are widely accepted as true (Nissen, Magidson, Gross, & Bergstrom, 2016). Figure 1 illustrates this with a toy simulation of a set of studies testing a difference between means from two conditions. If we have results from a series of experiments, three of which found a statistically significant difference and three of which did not, this provides fairly strong evidence that the difference is real (panel a). However, if we add a further four experiments that were not reported because results were null, the evidence cumulates in the opposite direction. Thus, omission of null studies can drastically alter our impression of the overall support for a hypothesis.

Since the paper by Simmons et al. (2011), there has been a dramatic increase in replication studies. As a result, a number of well-established phenomena in psychology have come into question. Often it is difficult to be certain whether the original reports were false positives, whether the replication was flawed, or whether the effect of interest is only evident under specific conditions—see, for example, Hobson and Bishop (2016) on mu suppression in response to observed actions; Sripada, Kesller, and Jonides (2016) on ego depletion; Lehtonen et al. (2018) on an advantage in cognitive control for bilinguals; O’Donnell et al. (2018) on the professor-priming effect; and Oostenbroek et al. (2016) on neonatal imitation. What is clear is that the size, robustness, and generalisability of many classic effects are lower than previously thought.

Selective reporting, through *p*-hacking and publication bias, is not the only blight on our science. A related problem is many editors place emphasis on reporting results in a way that “tells a good story,” even if that means retrofitting our hypothesis to the data, i.e., HARKing or “hypothesising after the results are known” (Kerr, 1998). Oberauer and Lewandowsky (2019) drew parallels between HARKing and *p*-hacking: in HARKing, there is post hoc selection of hypotheses, rather than selection of results or an analytic method. They proposed that HARKing is most widely used in fields where theories are so underspecified that they can accommodate many hypotheses and where there is a lack of “disconfirmatory diagnosticity,” i.e., failure to support a prediction is uninformative.

A lack of statistical power is a further problem for psychology—one that has been recognised since 1969, when Jacob Cohen exhorted psychologists not to waste time and effort doing experiments that had too few observations to show an effect of interest. In other fields, notably clinical trials and genetics, after a period where non-replicable results proliferated, underpowered studies died out quite rapidly when journals adopted stringent criteria for publication (e.g., Johnston, Lahey, & Matthys, 2013), and funders began to require power analysis in grant proposals. Psychology, however, has been slow to catch up.

It is not just experimental psychology that has these problems—studies attempting to link psychological traits and disorders to genetic and/or neurobiological variables are, if anything, subject to greater challenges. A striking example comes from a meta-analysis of links between the serotonin transporter gene, 5-HTTPLR, and depression. This postulated association has attracted huge research interest over the past 20 years, and the meta-analysis included 450 studies. Contrary to expectation, it concluded that there was no evidence of association. In a blog post summarising findings, Alexander (2019) wrote,

. . . what bothers me isn’t just that people said 5-HTTLPR mattered and it didn’t. It’s that we built whole imaginary edifices, whole castles in the air on top of this idea of 5-HTTLPR mattering. We “figured out” how 5-HTTLPR exerted its effects, what parts of the brain it was active in, what sorts of things it interacted with, how its effects were enhanced or suppressed by the effects of other imaginary depression genes. This isn’t just an explorer coming back from the Orient and claiming there are unicorns there. It’s the explorer describing the life cycle of unicorns, what unicorns eat, all the different subspecies of unicorn, which cuts of unicorn meat are tastiest, and a blow-by-blow account of a wrestling match between unicorns and Bigfoot.

It is no exaggeration to say that our field is at a crossroads (Pashler & Wagenmakers, 2012), and the 5-HTTLPR story is just a warning sign that practices that lead to bad science are widespread. If we continue to take the well-trodden path, using traditional methods for cooking data and asterisk hunting, we are in danger of losing attention, respect, and funding.

Much has been written about how we might tackle the so-called “replication crisis.” There have been four lines of attack. First, there have been calls for greater openness and transparency (Nosek et al., 2015). Second, a case has been made for better training in methods (e.g., Rousselet, Pernet, & Wilcox, 2017). Third, it has been argued we need to change the way research has been conducted to incorporate pre-registration of research protocols, preferably in the format of Registered Reports, which are peer-reviewed prior to data collection (Chambers, 2019). Fourth, it is recognised that for too long, the incentive structure of research has prioritised innovative, groundbreaking results over methodological quality. Indeed, Smaldino and McElreath (2016) suggested that one can model the success of scientists in a field as an evolutionary process, where prestigious publications lead to survival, leaving those whose work is less exciting to wither away and leave science. The common thread to these efforts is that they locate the mechanisms of bad science at the systemic level, in ways in which cultures and institutions reinforce norms and distribute resources. The solutions are, therefore, aimed at correcting these shortcomings by creating systems that make good behaviour easier and more rewarding and make poor behaviour more costly.

My view, however, is that institutional shortcomings are only part of the story: to improve scientific research, we also need to understand the mechanisms that maintain bad practices in individual humans. Bad science is usually done because somebody mistook it for good science. Understanding why individual scientists mistake bad science for good, and helping them to resist these errors, is a necessary component of the movement to improve psychology. I will argue that we need to understand how cognitive constraints lead to faulty reasoning if we are to get science back on course and persuade those who set the incentives to reform. Fortunately, as psychologists, we are uniquely well positioned to tackle this issue.

Experimental psychology has a rich tradition of studying human reasoning and decision-making, documenting the flaws and foibles that lead us to selectively process some types of information, make judgements on the basis of incomplete evidence, and sometimes behave in ways that seem frankly irrational. This line of work has had significant application to economics, politics, business studies, and law, but, with some notable exceptions (e.g., Hossenfelder, 2018; Mahoney, 1976), it has seldom been considered when studying the behaviour of research scientists. In what follows, I consider how our knowledge of human cognition can make sense of problematic scientific practices, and I propose ways we might use this information to find solutions.

### Cognitive constraints that affect how psychological science is done

Table 1 lists four characteristics of human cognition that I focus on: I refer to these as “constraints” because they limit how we process, understand, or remember information, but it is important to note that they include some biases that can be beneficial in many contexts. The first constraint is confirmation bias. As Hahn and Harris (2014) noted, a range of definitions of “confirmation bias” exist—here, I will define it as the tendency to seek out evidence that supports our position. A further set of constraints has to do with understanding of probability. A lack of an intuitive grasp of probability contributes to both neglect of statistical power in study design and *p*-hacking in data analysis. Third, there is an asymmetry in moral reasoning that can lead us to treat errors of omission as less culpable than errors of commission, even when their consequences are equally serious (Haidt & Baron, 1996). The final constraint featured in Bartlett’s (1932) work: reliance on cognitive schemata to fill in unstated information, leading to “reconstructive remembering,” which imbues memories with meaning while filtering out details that do not fit preconceptions.

**Table 1.** Different types of cognitive constraints.

Cognitive constraint	Description
Confirmation bias	Tendency to seek out and remember evidence that supports a preferred viewpoint
Misunderstanding of probability	(a) Failure to understand how estimation scales with sample size
	(b) Failure to understand that probability depends on context
Asymmetric moral reasoning	Errors of omission judged less seriously than errors of commission
Reliance on schemata	Perceiving and/or remembering in line with pre-existing knowledge, leading to omission or distortion of irrelevant information

In what follows, I illustrate how these constraints assume particular importance at different stages of the research process, as shown in Table 2.

**Table 2.** Cognitive constraints that operate at different stages of the research process.

Stage of research	Cognitive constraint
Experimental design	Confirmation bias: looking for evidence consistent with theory
	Statistical misunderstanding: power
Data analysis	Statistical misunderstanding: *p*-hacking
	Moral asymmetry: omission and “paltering” deemed acceptable
Scientific reporting	Confirmation bias in reviewing literature
	Moral asymmetry: omission and “paltering” deemed acceptable
	Cognitive schemata: need for narrative, HARKing

HARKing: hypothesising after the results are known.

## Bias in experimental design

### Confirmation bias and the failure to consider alternative explanations

Scientific discovery involves several phases: the researcher needs to (a) assemble evidence, (b) look for meaningful patterns and regularities in the data, (c) formulate a hypothesis, and (d) test it empirically by gathering informative new data. Steps (a)–(c) may be designated as exploratory and step (d) as hypothesis testing or confirmatory (Wagenmakers, Wetzels, Borsboom, van der Mass, & Kievit, 2012). Importantly, the same experiment cannot be used to both formulate and confirm a hypothesis. In practice, however, the distinction between the two types of experiment is often blurred.

Our ability to see patterns in data is vital at the exploratory stage of research: indeed, seeing something that nobody else has observed is a pinnacle of scientific achievement. Nevertheless, new ideas are often slow to be accepted, precisely because they do not fit the views of the time. One such example is described by Zilles and Amunts (2010): Brodmann’s cytoarchitectonic map of the brain, described in 1909. This has stood the test of time and is still used over 100 years later, but for several decades, it was questioned by those who could not see the fine distinctions made by Brodmann. Indeed, criticisms of poor reproducibility and lack of objectivity were levelled against him.

Brodmann’s case illustrates that we need to be cautious about dismissing findings that depend on special expertise or unique insight of the observer. However, there are plenty of other instances in the history of science where invalid ideas persisted, especially if proposed by an influential or charismatic figure. Entire edifices of pseudoscience have endured because we are very bad at discarding theories that do not work; as Bartlett (1932) would predict, new information that is consistent with the theory will strengthen its representation in our minds, but inconsistent information will be ignored. Examples from the history of science include the *rete mirabile*, a mass of intertwined arteries that is found in sheep but wrongly included in anatomical drawings of humans for over 1,000 years because of the significance attributed to this structure by Galen (Bataille et al., 2007); the planet Vulcan, predicted by Newton’s laws and seen by many astronomers until its existence was disproved by Einstein’s discoveries (Levenson, 2015); and N-rays, non-existent rays seen by at least 40 people and analysed in 3,090 papers by 100 scientists between 1903 and 1906 (Nye, 1980).

**Figure 2.**
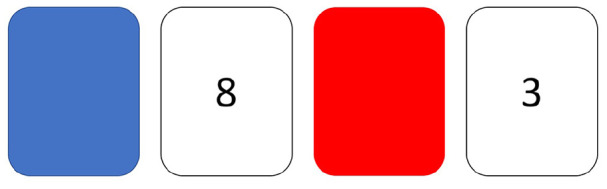
Wason’s (1960) task: The subject is told, “Each card has a number on one side and a patch of colour on the other. You are asked to test the hypothesis that—for these 4 cards—if an even number appears on one side, then the opposite side is red. Which card(s) would you turn over to test the hypothesis?”

Popper’s (1934/1959) goal was to find ways to distinguish science from pseudoscience, and his contribution to philosophy of science was to emphasise that we should be bold in developing ideas but ruthless in attempts to falsify them. In an early attempt to test scientists’ grasp of Popperian logic, Mahoney (1976) administered a classic task developed by Wason (1960) to 84 scientists (physicists, biologists, psychologists, and sociologists). In this deceptively simple task, people are shown four cards and told that each card has a number on one side and a patch of colour on the other side. The cards are placed to show number 3, number 8, red, and blue, respectively (see Figure 2). The task is to identify which cards need to be turned over to test the hypothesis that if an even number appears on one side, then the opposite side is red. The subject can pick any number of cards. The correct response is to name the two cards that could disconfirm the hypothesis—the number 8 and the blue card. Fewer than 10% of the scientists tested by Mahoney identified both critical cards, more often selecting the number 8 and the red card.

Although this study was taken as evidence of unscientific reasoning by scientists, that conclusion has since been challenged by those who have criticised both Popperian logic, in general, and the Wason selection task, in particular, as providing an unrealistic test of human rationality. For a start, the Wason task uses a deterministic hypothesis that can be disproved by a single piece of evidence. This is not a realistic model of biological or behavioural sciences, where we seldom deal with deterministic phenomena. Consider the claim that smoking causes lung cancer. Most of us accept that this is so, even though we know there are people who smoke and who do not get lung cancer and people who get lung cancer but never smoked. When dealing with probabilistic phenomena, a Bayesian approach makes more sense, whereby we consider the accumulated evidence to determine the relative likelihood of one hypothesis over another (as illustrated in Figure 1). Theories are judged as more or less probable, rather than true or false. Oaksford and Chater (1994) showed that, from a Bayesian perspective, typical selections made on the Wason task would be rational in contexts where the antecedent and consequent of the hypothesis (an even number and red colour) were both rare. Subsequently, Perfors and Navarro (2009) concluded that in situations where rules are relevant only for a minority of entities, then confirmation bias is an efficient strategy.

This kind of analysis has shifted the focus to discussions about how far, and under what circumstances, people are rational decision-makers. However, it misses a key point about scientific reasoning, which is that it involves an active process of deciding which evidence to gather, rather than merely a passive evaluation of existing evidence. It seems reasonable to conclude that, when presented with a particular set of evidence, people generally make decisions that are rational when evaluated against Bayesian standards. However, history suggests that we are less good at identifying which new evidence needs to be gathered to evaluate a theory. In particular, people appear to have a tendency to accept a hypothesis on the basis of “good enough” evidence, rather than actively seeking evidence for alternative explanations. Indeed, an early study by Doherty, Mynatt, Tweney, and Schiavo (1979) found that, when given an opportunity to select evidence to help decide which of two hypotheses was true (in a task where a fictitious pot had to be assigned as originating from one of the two islands that differed in characteristic features), people seemed unable to identify which information would be diagnostic and tended, instead, to select information that could neither confirm nor disconfirm their hypothesis.

Perhaps the strongest evidence for our poor ability to consider alternative explanations comes from the history of the development of clinical trials. Although James Lind is credited with doing the first trials for treatment of scurvy in 1747, it was only in 1948 that the randomised controlled trial became the gold standard for evaluating medical interventions (Vallier & Timmerman, 2008). The need for controls is not obvious, and people who are not trained in this methodology will often judge whether a treatment is effective on the basis of a comparison on an outcome measure between a pre-treatment baseline and a post-treatment evaluation. The logic is that if a group of patients given the treatment does not improve, the treatment did not work. If they do show meaningful gains, then it did work. And we can even embellish this comparison with a test of statistical significance. This reasoning can be seen as entirely rational, and this can explain why so many people are willing to accept that alternative medicine is effective.

The problem with this approach is that the pre–post intervention comparison allows important confounds to creep in. For instance, early years practitioners argue that we should identify language problems in toddlers so that we can intervene early. They find that if 18-month-old late talkers are given intervention, only a minority still have language problems at 2 years and, therefore, conclude the intervention was effective. However, if an untreated control group is studied over the same period, we find very similar rates of improvement (Wake et al., 2011)—presumably due to factors such a spontaneous resolution of problems or regression to the mean, which will lead to systematic bias in outcomes. Researchers need training to recognise causes of bias and to take steps to overcome them: thinking about possible alternative explanations of an observed phenomenon does not come naturally, especially when the preliminary evidence looks strong.

Intervention studies provide the clearest evidence of what I term “premature entrenchment” of a theory: some other examples are summarised in Table 3. Note that these examples do not involve poor replicability, quite the opposite. They are all cases where an effect, typically an association between variables, is reliably observed, and researchers then converge on accepting the most obvious causal explanation, without considering lines of evidence that might point to alternative possibilities.

**Table 3.** Premature entrenchment: examples where the most obvious explanation for an observed association is accepted for many years, without considering alternative explanations that could be tested with different evidence.

Observation	Favoured explanation	Alternative explanation	Evidence for alternative explanation
Home literacy environment predicts reading outcomes in children	Access to books at home affects children’s learning to read (Sénéchal & LeFevre, 2002)	Parents and children share genetic risk for reading problems	Children who are poor readers tend to have parents who are poor readers (Van Bergen, van Zuijen, Bishop, & de Jong, 2017)
Speech sounds (phonemes) do not have consistent auditory correlates but can be identified by knowledge of articulatory configurations used to produce them	Motor theory of speech perception: we learn to recognise speech by mapping input to articulatory gestures (Liberman, Cooper, Shankweiler, & Studdert-Kennedy, 1967)	Correlations between perception and production reflect co-occurrence rather than causation	Children who are congenitally unable to speak can develop good speech perception, despite having no articulatory experience (Bishop & Robson, 1989)
Dyslexics have atypical brain responses to speech when assessed using fMRI	Atypical brain organisation provides evidence that dyslexia is a “real disorder” with a neurobiological basis (Shaywitz, Mody, & Shaywitz, 2006)	Atypical responses to speech in the brain are a consequence of being a poor reader	Adults who had never been taught to read have atypical brain organisation for spoken language (Dehaene et al., 2010)

fMRI: functional magnetic resonance imaging.

Premature entrenchment may be regarded as evidence that humans adopt Bayesian reasoning: we form a prior belief about what is the case and then require considerably more evidence to overturn that belief than to support it. This would explain why, when presented with virtually identical studies that either provided support for or evidence against astrology, psychologists were more critical of the latter (Goodstein & Brazis, 1970). The authors of that study expressed concern about the “double standard” shown by biased psychologists who made unusually harsh demands of research in borderline areas, but from a Bayesian perspective, it is reasonable to use prior knowledge so that extraordinary claims require extraordinary evidence. Bayesian reasoning is useful in many situations: it allows us to act decisively on the basis of our long-term experience, rather than being swayed by each new incoming piece of data. However, it can be disastrous if we converge on a solution too readily on the basis of incomplete or inaccurate information. This will be exacerbated by publication bias, which distorts the evidential landscape.

For many years, the only methods available to counteract the tendency for premature entrenchment were exhortations to be self-critical (e.g., Feynman, 1974) and peer review. The problem with peer review is that it typically comes too late to be useful, after research is completed. In the final section of this article, I will consider some alternative approaches that bring in external appraisal of experimental designs at an earlier stage in the research process.

### Misunderstanding of probability leading to underpowered studies

Some 17 years after Cohen’s seminal work on statistical power, Newcombe (1987) wrote,

Small studies continue to be carried out with little more than a blind hope of showing the desired effect. Nevertheless, papers based on such work are submitted for publication, especially if the results turn out to be statistically significant. (p. 657)

In clinical medicine, things have changed, and the importance of adequate statistical power is widely recognised among those conducting clinical trials. But in psychology, the “blind hope” has persisted, and we have to ask ourselves why this is.

My evidence here is anecdotal, but the impression is that many psychologists simply do not believe advice about statistical power, perhaps because there are so many underpowered studies published in the literature. When a statistician is consulted about sample size for a study, he or she will ask the researcher to estimate the anticipated effect size. This usually leads to a sample size estimate that is far higher than the researcher anticipated or finds feasible, leading to a series of responses not unlike the first four of the five stages of grief: denial, anger, bargaining, and depression. The final stage, acceptance, may, however, not be reached.

Of course, there are situations where small sample sizes are perfectly adequate: the key issue is how large the effect of interest is in relation to the variance. In some fields, such as psychophysics, you may not even need statistics—the famous “interocular trauma” test (referring to a result so obvious and clear-cut that it hits you between the eyes) may suffice. Indeed, in such cases, recruitment of a large sample would just be wasteful.

There are, however, numerous instances in psychology where people have habitually used sample sizes that are too small to reliably detect an effect of interest: see, for instance, the analysis by Poldrack et al. (2017) of well-known effects in functional magnetic resonance imaging (fMRI) or Oakes (2017) on looking-time experiments in infants. Quite often, a line of research is started when a large effect is seen in a small sample, but over time, it becomes clear that this is a case of “winner’s curse,” a false positive that is published precisely because it looks impressive but then fails to replicate when much larger sample sizes are used. There are some recent examples from studies looking at neurobiological or genetic correlates of individual differences, where large-scale studies have failed to support previously published associations that had appeared to be solid (e.g., De Kovel & Francks, 2019, on genetics of handedness; Traut et al., 2018, on cerebellar volume in autism; Uddén et al., 2019, on genetic correlates of fMRI language-based activation).

A clue to the persistence of underpowered psychology studies comes from early work by Tversky and Kahneman (1971, 1974). They studied a phenomenon that they termed “belief in the law of small numbers,” an exaggerated confidence in the validity of conclusions based on small samples, and showed that even those with science training tended to have strong intuitions about random sampling that were simply wrong. They illustrated this with the following problem:

A certain town is served by two hospitals. In the larger hospital about 45 babies are born each day, and in the smaller hospital about 15 babies are born each day. As you know, about 50% of all babies are boys. However, the exact percentage varies from day to day. Sometimes it may be higher than 50%, sometimes lower. For a period of 1 year, each hospital recorded the days on which more than 60% of the babies born were boys. Which hospital do you think recorded more such days?

1. The large hospital

2. The small hospital

3. About the same (that is, within 5% of each other)

**Figure 3.**
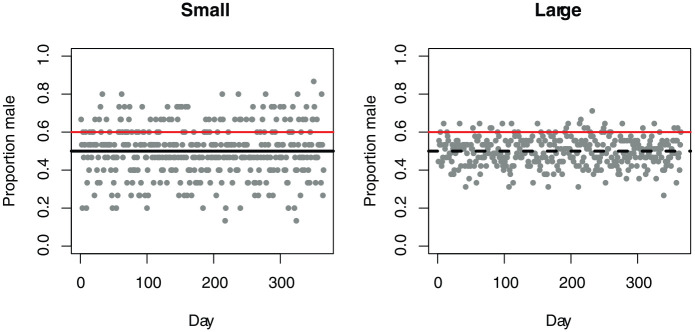
Simulated data showing proportions of males born in a small hospital with 15 births per day versus a large hospital with 45 births per day. The small hospital has more days where more than 60% of births are boys (points above red line).

Most people selected Option 3, whereas, as illustrated in Figure 3, Option 2 is the correct answer—with only 15 births per day, the day-to-day variation in the proportion of boys will be much higher than with 45 births per day, and hence, more days will have more than 60% boys. One reason why our intuitions deceive us is because the sample size does not affect the average percentage of male births in the long run: this will be 50%, regardless of the hospital size. But sample size has a dramatic impact on the variability in the proportion of male births from day to day. More formally, if you have a big and small sample drawn from the same population, the expected estimate of the mean will be the same, but the standard error of that estimate will be greater for the small sample.

Statistical power depends on the effect size, which, for a simple comparison of two means, can be computed as the difference in means divided by the pooled standard deviation. It follows that power is crucially dependent on the proportion of variance in observations that is associated with an effect of interest, relative to background noise. Where variance is high, it is much harder to detect the effect, and hence, small samples are often underpowered. Increasing the sample size is not the only way to improve power: other options include improving the precision of measurement, using more effective manipulations, or adopting statistical approaches to control noise (Lazic, 2018). But in many situations, increasing the sample size is the preferred approach to enhance statistical power to detect an effect.

## Bias in data analysis: *p*-hacking

*P*-hacking can take various forms, but they all involve a process of selective analysis. Suppose some researchers hypothesise that there is an association between executive function and implicit learning in a serial reaction time task, and they test this in a study using four measures of executive function. Even if there is only one established way of scoring each task, they have four correlations; this means that the probability that none of the correlations is significant at the .05 level is .95^4^—i.e., .815—and conversely, the probability that at least one is significant is .185. This probability can be massaged to even higher levels if the experimenters look at the data and then select an analytic approach that maximises the association: maybe by dropping outliers, by creating a new scoring method, combining measures in composites, and so on. Alternatively, the experimenters may notice that the strength of the correlation varies with the age or sex of participants and so subdivide the sample to coax at least a subset of data into significance. The key thing about *p*-hacking is that at the end of the process, the researchers selectively report the result that “worked,” with the implication that the *p*-value can be interpreted at face value. But it cannot: probability is meaningless if not defined in terms of a particular analytic context. *P*-hacking appears to be common in psychology (John, Loewenstein, & Prelec, 2012). I argue here that this is because it arises from a conjunction of two cognitive constraints: failure to understand probability, coupled with a view that omission of information when reporting results is not a serious misdemeanour.

### Failure to understand probability

In an influential career guide, published by the American Psychological Association, Bem (2004) explicitly recommended going against the “conventional view” of the research process, as this might lead us to miss exciting new findings. Instead readers were encouraged to

become intimately familiar with . . . the data. Examine them from every angle. Analyze the sexes separately. Make up new composite indexes. If a datum suggests a new hypothesis, try to find additional evidence for it elsewhere in the data. If you see dim traces of interesting patterns, try to reorganize the data to bring them into bolder relief. If there are participants you don’t like, or trials, observers, or interviewers who gave you anomalous results, drop them (temporarily). Go on a fishing expedition for something—anything—interesting. (p. 2)

For those who were concerned this might be inappropriate, Bem offered reassurance. Everything is fine because what you are doing is exploring your data. Indeed, he implied that anyone who follows the “conventional view” would be destined to do boring research that nobody will want to publish.

Of course, Bem (2004) was correct to say that we need exploratory research. The problem comes when exploratory research is repackaged as if it were hypothesis testing, with the hypothesis invented after observing the data (HARKing), and the paper embellished with *p*-values that are bound to be misleading because they were *p*-hacked from numerous possible values, rather than derived from testing an a priori hypothesis. If results from exploratory studies were routinely replicated, prior to publication, we would not have a problem, but they are not. So why did the American Psychological Association think it appropriate to publish Bem’s views as advice to young researchers? We can find some clues in the book overview, which explains that there is a distinction between the “formal” rules that students are taught and the “implicit” rules that are applied in everyday life, concluding that “This book provides invaluable guidance that will help new academics plan, play, and ultimately win the academic career game.” Note that the stated goal is not to do excellent research: it is to have “a lasting and vibrant career.” It seems, then, that there is recognition here that if you do things in the “conventional” way, your career will suffer. It is clear from Bem’s framing of his argument that he was aware that his advice was not “conventional,” but he did not think it was unethical—indeed, he implied it would be unfair on young researchers to do things conventionally as that will prevent them making exciting discoveries that will enable them to get published and rise up the academic hierarchy. While it is tempting to lament the corruption of a system that treats an academic career as a game, it is more important to consider why so many people genuinely believe that *p*-hacking is a valid, and indeed creative, approach to doing research.

The use of null-hypothesis significance testing has attracted a lot of criticism, with repeated suggestions over the years that *p*-values be banned. I favour the more nuanced view expressed by Lakens (2019), who suggests that *p*-values have a place in science, provided they are correctly understood and used to address specific questions. There is no doubt, however, that many people do misunderstand the *p*-value. There are many varieties of misunderstanding, but perhaps the most common is to interpret the *p*-value as a measure of strength of evidence that can be attached to a given result, regardless of the context. It is easy to see how this misunderstanding arises: if we hold the sample size constant, then for a single comparison, there will be a linear relationship between the *p*-value and the effect size. However, whereas an effect size remains the same, regardless of the analytic context, a *p*-value is crucially context-dependent.

Suppose in the fictitious study of executive function described above, the researchers have 20 participants and four measures of executive function (A–D) that correlate with implicit learning with *r* values of .21, .47, .07, and −.01. The statistics package tells us that the corresponding two-tailed *p*-values are .374, .037, .769, and .966. A naive researcher may rejoice at having achieved significance with the second correlation. However, as noted above, the probability that at least one correlation of the four will have an associated *p*-value of less than .05 is 18%, not 5%. If we want to identify correlations that are unlikely under the null hypothesis, then we need to correct the alpha level (e.g., by doing a Bonferroni correction to adjust by the number of tests, i.e., .05/4 = .0125). At this point, the researchers see their significant result snatched from their grasp. This creates a strong temptation to just drop the three non-significant tests and not report them. Alternatively, one sometimes sees papers that report the original *p*-value but then state that it “did not survive” Bonferroni correction, but they, nevertheless, exhume it and interpret the uncorrected value. Researchers acting this way may not think that they are doing anything inappropriate, other than going against advice of pedantic statisticians, especially given Bem’s (2004) advice to follow the “implicit” rather than “formal” rules of research. However, this is simply wrong: as illustrated above, a *p*-value can only be interpreted in relation to the context in which it is computed.

One way of explaining the notion of *p*-hacking is to use the old-fashioned method of games of chance. I find this scenario helpful: we have a magician who claims he can use supernatural powers to deal a poker hand of “three of a kind” from an unbiased deck of cards. This type of hand will occur in around 1 of 50 draws from an unbiased deck. He points you to a man who, to his amazement, finds that his hand contains three of a kind. However, you then discover he actually tried his stunt with 50 people, and this man was the only one who got three of a kind. You are rightly disgruntled. This is analogous to *p*-hacking. The three-of-a-kind hand is real enough, but its unusualness, and hence its value as evidence of the supernatural, depends on the context of how many tests were done. The probability that needs to be computed here is not the probability of one specific result but rather the probability that specific result would come up at least once in 50 trials.

### Asymmetry of sins of omission and commission

According to Greenwald (1975) “[I]t is a truly gross ethical violation for a researcher to suppress reporting of difficult-to-explain or embarrassing data to present a neat and attractive package to a journal editor” (p. 19).

However, this view is not universal.

Greenwald’s focus was on publication bias, i.e., failure to report an entire study, but the point he made about “prejudice” against null results also applies to cases of *p*-hacking where only “significant” results are reported, whereas others go unmentioned. It is easy to see why scientists might play down the inappropriateness of *p*-hacking, when it is so important to generate “significant” findings in a world with a strong prejudice against null results. But I suspect another reason why people tend to underrate the seriousness of *p*-hacking is because it involves an error of omission (failing to report the full context of a *p*-value), rather than an error of commission (making up data).

In studies of morality judgement, errors of omission are generally regarded as less culpable than errors of commission (see, e.g., Haidt & Baron, 1996). Furthermore, *p*-hacking may be seen to involve a particularly subtle kind of dishonesty because the statistics and their associated *p*-values are provided by the output of statistics software. They are mathematically correct when testing a specific, prespecified hypothesis: the problem is that, without the appropriate context, they imply stronger evidence than is justified. This is akin to what Rogers, Zeckhauser, Gino, Norton, and Schweitzer (2017) have termed “paltering,” i.e., the use of truthful statements to mislead, a topic they studied in the context of negotiations. An example was given of a person trying to sell a car that had twice needed a mechanic to fix it. Suppose the potential purchaser directly asks “Has the car ever had problems?” An error of commission is to deny the problems, but a paltering answer would be “This car drives very smoothly and is very responsive. Just last week it started up with no problems when the temperature was −5 degrees Fahrenheit.” Rogers et al. showed that negotiators were more willing to palter than to lie, although potential purchasers regarded paltering as only marginally less immoral than lying.

Regardless of the habitual behaviour of researchers, the general public does not find *p*-hacking acceptable. Pickett and Roche (2018) did an M-Turk experiment in which a community sample was asked to judge the morality of various scenarios, including this one:

A medical researcher is writing an article testing a new drug for high blood pressure. When she analyzes the data with either method A or B, the drug has zero effect on blood pressure, but when she uses method C, the drug seems to reduce blood pressure. She only reports the results of method C, which are the results that she wants to see.

Seventy-one percent of respondents thought this behaviour was immoral, 73% thought the researcher should receive a funding ban, and 63% thought the researcher should be fired.

Nevertheless, although selective reporting was generally deemed immoral, data fabrication was judged more harshly, confirming that in this context, as in those studied by Haidt and Baron (1996), sins of commission are taken more seriously than errors of omission.

If we look at the consequences of a specific act of *p*-hacking, it can potentially be more serious than an act of data fabrication: this is most obvious in medical contexts, where suppression of trial results, either by omitting findings from within a study or by failing to publish studies with null results, can provide a badly distorted basis for clinical decision-making. In their simulations of evidence cumulation, Nissen et al. (2016) showed how *p*-hacking could compound the impact of publication bias and accelerate the premature “canonization” of theories; the alpha level that researchers assume applies to experimental results is distorted by *p*-hacking, and the expected rate of false positives is actually much higher. Furthermore, *p*-hacking is virtually undetectable because the data that are presented are real, but the necessary context for their interpretation is missing. This makes it harder to correct the scientific record.

## Bias in writing up a study

Most writing on the “replication crisis” focuses on aspects of experimental design and observations, data analysis, and scientific reporting. The resumé of literature that is found in the introduction to empirical papers, as well as in literature review articles, is given less scrutiny. I will make the case that biased literature reviews are universal and have a major role in sustaining poor reproducibility because they lead to entrenchment of false theories, which are then used as the basis for further research.

It is common to see biased literature reviews that put a disproportionate focus on findings that are consistent with the author’s position. Researchers who know an area well may be aware of this, especially if their own work is omitted, but in general, cherry-picking of evidence is hard to detect. I will use a specific paper published in 2013 to illustrate my point, but I will not name the authors, as it would be invidious to single them out when the kinds of bias in their literature review are ubiquitous. In their paper, my attention was drawn to the following statement in the introduction:

Regardless of etiology, cerebellar neuropathology commonly occurs in autistic individuals. Cerebellar hypoplasia and reduced cerebellar Purkinje cell numbers are the most consistent neuropathologies linked to autism. … MRI studies report that autistic children have smaller cerebellar vermal volume in comparison to typically developing children.

I was surprised to read this because a few years ago, I had attended a meeting on neuroanatomical studies of autism and had come away with the impression that there were few consistent findings. I did a quick search for an up-to-date review, which turned up a meta-analysis (Traut et al., 2018), that included 16 MRI studies published between 1997 and 2010, five of which reported larger cerebellar size in autism and one of which found smaller cerebellar size. In the article I was reading, one paper had been cited to support the MRI statement, but it referred to a study where the absolute size of the vermis did not differ from typically developing children but was *relatively* small in the autistic participants, after the overall (larger) size of the cerebellum had been controlled for.

Other papers cited to support the claims of cerebellar neuropathology included a couple of early post mortem neuroanatomical studies, as well as two reviews. The first of these (DiCicco-Bloom et al., 2006) summarised presentations from a conference and supported the claims made by the authors. The other one, however (Palmen, van Engeland, Hof, & Schmitz, 2004), expressed more uncertainty and noted a lack of correspondence between early neuroanatomical studies and subsequent MRI findings, concluding,

Although some consistent results emerge, the majority of the neuropathological data remain equivocal. This may be due to lack of statistical power, resulting from small sample sizes and from the heterogeneity of the disorder itself, to the inability to control for potential confounding variables such as gender, mental retardation, epilepsy and medication status, and, importantly, to the lack of consistent design in histopathological quantitative studies of autism published to date.

In sum, a confident statement “cerebellar neuropathology commonly occurs in autistic individuals,” accompanied by a set of references, converged to give the impression that there is consensus that the cerebellum is involved in autism. However, when we drill down, we find that the evidence is uncertain, with discrepancies between neuropathological studies and MRI and methodological concerns about the former. Meanwhile, this study forms part of a large body of research in which genetically modified mice with cerebellar dysfunction are used as an animal model of autism. My impression is that few of the researchers using these mouse models appreciate that the claim of cerebellar abnormality in autism is controversial among those working with humans because each paper builds on the prior literature. There is entrenchment of error, for two reasons. First, many researchers will take at face value the summary of previous work in a peer-reviewed paper, without going back to original cited sources. Second, even if a researcher is careful and scholarly and does read the cited work, they are unlikely to find relevant studies that were not included in the literature review.

It is easy to take an example like this and bemoan the lack of rigour in scientific writing, but this is to discount cognitive biases that make it inevitable that, unless we adopt specific safeguards against this, cherry-picking of evidence will be the norm. Three biases lead us in this direction: confirmation bias, moral asymmetry, and reliance on schemata.

### Confirmation bias: cherry-picking prior literature

A personal example may serve to illustrate the way confirmation bias can operate subconsciously. I am interested in genetic effects on children’s language problems, and I was in the habit of citing three relevant twin studies when I gave talks on this topic. All these obtained similar results, namely that there was a strong genetic component to developmental language disorders, as evidenced by much higher concordance for disorder in pairs of monozygotic versus dizygotic twins. In 2005, however, Hayiou-Thomas, Oliver, and Plomin published a twin study with very different findings, with low twin/co-twin concordance, regardless of zygosity. It was only when I came to write a review of this area and I checked the literature that I realised I had failed to mention the 2005 study in talks for a year or two, even though I had collaborated with the authors and was well aware of the findings. I had formed a clear view on heritability of language disorders, and so I had difficulty remembering results that did not agree. Subsequently, I realised we should try to understand why this study obtained different results and found a plausible explanation (Bishop & Hayiou-Thomas, 2008). But I only went back for a further critical look at the study because I needed to make sense of the conflicting results. It is inevitable that we behave this way as we try to find generalisable results from a body of work, but it creates an asymmetry of attention and focus between work that we readily accept, because it fits, and work that is either forgotten or looked at more critically, because it does not.

**Figure 4.**
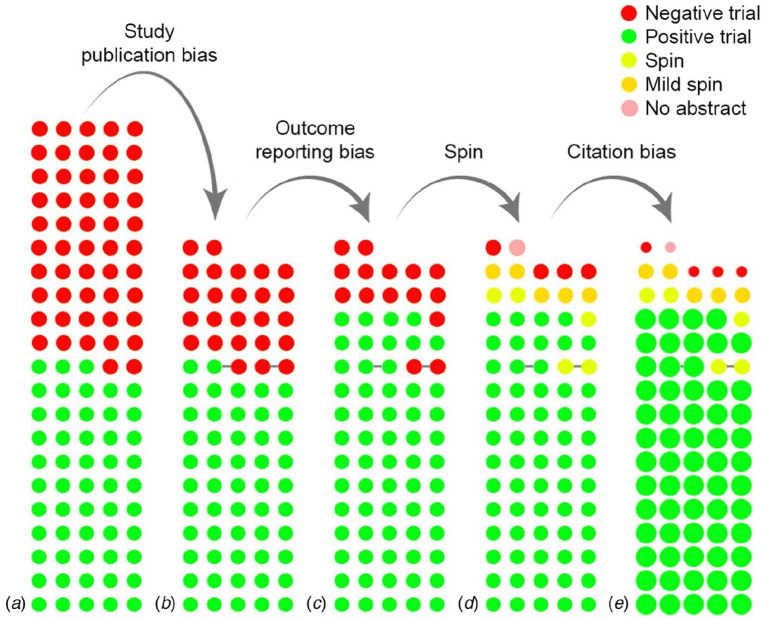
The cumulative impact of reporting and citation biases on the evidence base for antidepressants. (a) Displays the initial, complete cohort of trials that were recorded in a registry, while (b) through (e) show the cumulative effect of biases. Each circle indicates a trial, while the colour indicates whether results were positive or negative or were reported to give a misleadingly positive impression(spin). Circles connected by a grey line indicate trials from the same publication. The progression from (a) to (b) shows that nearly all the positive trials but only half of those with null results were published, and reporting of null studies showed (c) bias or (d) spin in what was reported. In (e), the size of the circle indicates the (relative) number of citations received by that category of studies. *Source.* Reprinted with permission from De Vries et al. (2018).

A particularly rich analysis of citation bias comes from a case study by Greenberg (2009), who took as his starting point papers concerned with claims that a protein, β amyloid, was involved in causing a specific form of muscle disease. Greenberg classified papers according to whether they were positive, negative, or neutral about this claim and carried out a network analysis to identify influential papers (those with many citations). He found that papers that were critical of the claim received far fewer citations than those that supported it, and this was not explained by lower quality. Animal model studies were almost exclusively justified by selective citation of positive studies. Consistent with the idea of “reconstructive remembering,” he also found instances where cited content was distorted, as well as cases where influential review papers amplified citation bias by focusing attention only on positive work. The net result was an information (perhaps better termed a disinformation) cascade that would lead to a lack of awareness of critical data, which never gets recognised. In effect, when we have agents that adopt Bayesian reasoning, if they are presented with distorted information, this creates a positive feedback loop that leads to increasing bias in the prior. Viewed this way, we can start to see how omission of relevant citations is not a minor peccadillo but a serious contributor to entrenchment of error. Further evidence of the cumulative impact of citation bias is shown in Figure 4, which uses studies of intervention for depression. Because studies in this area are registered, it is possible to track the fate of unpublished as well as published studies. The researchers showed that studies with null results are far less likely to be published than those with positive findings, but even if the former are published, there is a bias against citing them.

While describing such cases of citation bias, it is worth pausing to consider one of the best-known examples of distorted thinking: experimenter bias. This is similar to confirmation bias, but rather than involving selective attention to specific aspects of a situation that fits with our preconceptions, it has a more active character, whereby the experimenter can unwittingly influence the outcome of a study. The best-known research on this topic was the original Rosenthal and Fode (1963) study, where students were informed that the rats they were studying were “maze-bright” or “maze-dull,” when in fact they did not differ. Nevertheless, the “maze-bright” group learned better, suggesting that the experimenter would try harder to train an animal thought to have potential. A related study by Rosenthal and Jacobson (1963) claimed that if teachers were told that a test had revealed that specific pupils were “ready to bloom,” they would do better on an IQ test administered at the end of the year, even though the children so designated were selected at random.

Both these studies are widely cited. It is less well known that work on experimenter bias was subjected to a scathing critique by Barber and Silver (1968), entitled “Fact, fiction and the experimenter bias effect,” in which it was noted that work in this area suffered from poor methodological quality, in particular *p*-hacking. Barber and Silver did not deny that experimenter bias could affect results, but they concluded that these effects were far less common and smaller in magnitude than those implied by Rosenthal’s early work. Subsequently, Barber (1976) developed this critique further in his book Pitfalls in Human Research. Yet Rosenthal’s work is more highly cited and better remembered than that of Barber.

Rosenthal’s work provides a cautionary tale: although confirmation bias helps explain distorted patterns of citation, the evidence for maladaptive cognitive biases has been exaggerated. Furthermore, studies on confirmation bias often use artificial experiments, divorced from real life, and the criteria for deciding that reasoning is erroneous are often poorly justified (Hahn & Harris, 2014). In future, it would be worthwhile doing more naturalistic explorations of people’s memory for studies that do and do not support a position when summarising scientific literature.

On a related point, in using confirmation bias as an explanation for persistence of weak theories, there is a danger that I am falling into exactly the trap that I am describing. For instance, I was delighted to find Greenberg’s (2009) paper, as it chimed very well with my experiences when reading papers about cerebellar deficits in autism. But would I have described and cited it here if it had shown no difference between citations for papers that did and did not support the β amyloid claim? Almost certainly not. Am I going to read all literature on citation bias to find out how common it is? That strategy would soon become impossible if I tried to do it for every idea I touch upon in this article.

### Moral asymmetry between errors of omission and commission

The second bias that fortifies the distortions in a literature review is the asymmetry of moral judgement that I referred to when discussing *p*-hacking. To my knowledge, paltering has not been studied in the context of literature reviews, but my impression is that selective presentation of results that fit, while failing to mention important contextual factors (e.g., the vermis in those with autism is smaller but only when you have covaried for the total cerebellar size), is common. How far this is deliberate or due to reconstructive remembering, however, is impossible to establish.

It would also be of interest to conduct studies on people’s attitudes to the acceptability of cherry-picking of literature versus paltering (misleadingly selective reporting) or invention of a study. I would anticipate that most would regard cherry-picking as fairly innocuous, for several reasons: first, it could be an unintended omission; second, the consequences of omitting material from a review may be seen as less severe than introducing misinformation; and third, selective citation of papers that fit a narrative can have a positive benefit in terms of readability. There are also pragmatic concerns: some journals limit the word count for an introduction or reference list so that full citation of all relevant work is not possible and, finally, sanctioning people for harmful omissions would create apparently unlimited obligations (Haidt & Baron, 1996). Quite simply, there is far too much literature for even the most diligent scholar to read.

Nevertheless, consequences of omission can be severe. The above examples of research on the serotonin transporter gene in depression, or cerebellar abnormality in autism, emphasise how failure to cite earlier null results can lead to a misplaced sense of confidence in a phenomenon, which is wasteful in time and money when others attempt to build on it. And the more we encounter a claim, the more likely it is to be judged as true, regardless of actual accuracy (see Pennycook, Cannon, & Rand, 2018, for a topical example). As Ingelfinger (1976) put it, “faulty or inappropriate references . . . like weeds, tend to reproduce themselves and so permit even the weakest of allegations to acquire, with repeated citation, the guise of factuality” (p. 1076).

### Reliance on schemata

Our brains cannot conceivably process all the information around us: we have to find a way to select what is important to function and survive. This involves a search for meaningful patterns, which once established, allow us to focus on what is relevant and ignore the rest. Scientific discovery may be seen as an elevated version of pattern discovery: we see the height of scientific achievement as discovering regularities in nature that allow us to make better predictions about how the world behaves and to create new technologies and interventions from the basic principles we have discovered.

Scientific progress is not a simple process of weighing up competing pieces of evidence in relation to a theory. Rather than simply choosing between one hypothesis and another, we try to understand a problem in terms of a schema. Bartlett (1932) was one of the first psychologists to study how our preconceptions, or schemata, create distortions in perception and memory. He introduced the idea of “reconstructive remembering,” demonstrating how people’s memory of a narrative changed over time in specific ways, to become more coherent and aligned with pre-existing schemata.

Bartlett’s (1932) work on reconstructive remembering can explain why we not only tend to ignore inconsistent evidence (Duyx, Urlings, Swaen, Bouter, & Zeegers, 2017) but also are prone to distort the evidence that we do include (Vicente & Brewer, 1993). If we put together the combined influence of confirmation bias and reconstructive remembering, it suggests that narrative literature reviews have a high probability of being inaccurate: both types of bias will lead to a picture of research converging on a compelling story, when the reality may be far less tidy (Katz, 2013).

I have focused so far on bias in citing prior literature, but schemata also influence how researchers go about writing up results. If we just were to present a set of facts that did not cohere, our work would be difficult to understand and remember. As Chalmers, Hedges, and Cooper (2002) noted, this point was made in 1885 by Lord Raleigh in a presidential address to the British Association for the Advancement of Science:

If, as is sometimes supposed, science consisted in nothing but the laborious accumulation of facts, it would soon come to a standstill, crushed, as it were, under its own weight. The suggestion of a new idea, or the detection of a law, supersedes much that has previously been a burden on the memory, and by introducing order and coherence facilitates the retention of the remainder in an available form. (Rayleigh, 1885, p. 20)

Indeed, when we write up our research, we are exhorted to “tell a story,” which achieves the “order and coherence” that Rayleigh described. Since his time, ample literature on narrative comprehension has confirmed that people fill in gaps in unstated information and find texts easier to comprehend and memorise when they fit a familiar narrative structure (Bower & Morrow, 1990; Van den Broek, 1994).

This resonates with Dawkins’ (1976) criteria for a meme, i.e., an idea that persists by being transmitted from person to person. Memes need to be easy to remember, understand, and communicate, and so narrative accounts make far better memes than dry lists of facts. From this perspective, narrative serves a useful function in providing a scaffolding that facilitates communication. However, while this is generally a useful, and indeed essential, aspect of human cognition, in scientific communication, it can lead to propagation of false information. Bartlett (1932) noted that remembering is hardly ever really exact, “and it is not at all important that it should be so.” He was thinking of the beneficial aspects of schemata, in allowing us to avoid information overload and to focus on what is meaningful. However, as Dawkins emphasised, survival of a meme does not depend on it being useful or true. An idea such as the claim that vaccination causes autism is a very effective meme, but it has led to resurgence of diseases that were close to being eradicated.

In communicating scientific results, we need to strike a fine balance between presenting a precis of findings that is easily communicated and moving towards an increase in knowledge. I would argue the pendulum may have swung too far in the direction of encouraging researchers to tell good narratives. Not just media outlets, but also many journal editors and reviewers, encourage authors to tell simple stories that are easy to understand, and those who can produce these may be rewarded with funding and promotion.

The clearest illustration of narrative supplanting accurate reporting comes from the widespread use of HARKing, which was encouraged by Bem (2004) when he wrote,

There are two possible articles you can write: (a) the article you planned to write when you designed your study or (b) the article that makes the most sense now that you have seen the results. They are rarely the same, and the correct answer is (b).

Of course, formulating a hypothesis on the basis of observed data is a key part of the scientific process. However, as noted above, it is not acceptable to use the same data to both formulate and test the hypothesis—replication in a new sample is needed to avoid being misled by the play of chance and littering literature with false positives (Lazic, 2016; Wagenmakers et al., 2012).

Kerr (1998) considered why HARKing is a successful strategy and pointed out that it allowed the researcher to construct an account of an experiment that fits a good story script:

Positing a theory serves as an effective “initiating event.” It gives certain events significance and justifies the investigators’ subsequent purposeful activities directed at the goal of testing the hypotheses. And, when one HARKs, a “happy ending” (i.e., confirmation) is guaranteed. (p. 203)

In this regard, Bem’s advice makes perfect sense: “A journal article tells a straightforward tale of a circumscribed problem in search of a solution. It is not a novel with subplots, flashbacks, and literary allusions, but a short story with a single linear narrative line.”

We have, then, a serious tension in scientific writing. We are expected to be scholarly and honest, to report all our data and analyses and not to hide inconvenient truths. At the same time, if we want people to understand and remember our work, we should tell a coherent story from which unnecessary details have been expunged and where we cut out any part of the narrative that distracts from the main conclusions.

Kerr (1998) was clear that HARKing has serious costs. As well as translating type I errors into hard-to-eradicate theory, he noted that it presents a distorted view of science as a process which is far less difficult and unpredictable than is really the case. We never learn what did not work because inconvenient results are suppressed. For early career researchers, it can lead to cynicism when they learn that the rosy picture portrayed in the literature was achieved only by misrepresentation.

## Overcoming cognitive constraints to do better science

One thing that is clear from this overview is that we have known about cognitive constraints for decades, yet they continue to affect scientific research. Finding ways to mitigate the impact of these constraints should be a high priority for experimental psychologists. Here, I draw together some general approaches that might be used to devise an agenda for research improvement. Many of these ideas have been suggested before but without much consideration of cognitive constraints that may affect their implementation. Some methods, such as training, attempt to overcome the constraints directly in individuals: others involve making structural changes to how science is done to counteract our human tendency towards unscientific thinking. None of these provides a total solution: rather, the goal is to tweak the dials that dictate how people think and behave, to move us closer to better scientific practices.

### Training

It is often suggested that better training is needed to improve replicability of scientific results, yet the focus tends to be on formal instruction in experimental design and statistics. Less attention has been given to engendering a more intuitive understanding of probability, or counteracting cognitive biases, though there are exceptions, such as the course by Steel, Liermann, and Guttorp (2018), which starts with a consideration of “How the wiring of the human brain leads to incorrect conclusions from data.” One way of inducing a more intuitive sense of statistics and *p*-values is by using data simulations. Simulation is not routinely incorporated in statistics training, but free statistical software now makes this within the grasp of all (Tintle et al., 2015). This is a powerful way to experience how easy it is to get a “significant” *p*-value when running multiple tests. Students are often surprised when they generate repeated runs of a correlation matrix of random numbers with, say, five variables and find at least one “significant” correlation in about one in four runs. Once you understand that there is a difference between the probability associated with getting a specific result on a single test, predicted in advance, versus the probability of that result coming up at least once in a multitude of tests, then the dangers of *p*-hacking become easier to grasp.

Data simulation could also help overcome the misplaced “belief in the law of small numbers” (Tversky & Kahneman, 1974). By generating datasets with a known effect size, and then taking samples from these and subjecting them to statistical test, the student can learn to appreciate just how easy it is to miss a true effect (type II error) if the study is underpowered.

There is small literature evaluating attempts to specifically inoculate people against certain types of cognitive bias. For instance, Morewedge et al. (2015) developed instructional videos and computer games designed to reduce a series of cognitive biases, including confirmation bias, and found these to be effective over the longer term. Typically, however, such interventions focus on hypothetical scenarios outside the scope of experimental psychology. They might improve scientific quality of research projects if adjusted to make them relevant to conducting and appraising experiments.

### Triangulation of methods in study design

I noted above that for science to progress, we need to overcome a tendency to settle on the first theory that seems “good enough” to account for observations. Any method that forces the researcher to actively search for alternative explanations is, therefore, likely to stimulate better research.

The notion of triangulation (Munafò & Davey Smith, 2018) was developed in the field of epidemiology, where reliance is primarily on observational data, and experimental manipulation is not feasible. Inferring causality from correlational data is hazardous, but it is possible to adopt a strategic approach of combining complementary approaches to analysis, each of which has different assumptions, strengths, and weaknesses. Epidemiology progresses when different explanations for correlational data are explicitly identified and evaluated, and converging evidence is obtained (Lawlor, Tilling, & Davey Smith, 2016). This approach could be extended to other disciplines, by explicitly requiring researchers to use at least two different methods with different potential biases when evaluating a specific hypothesis.

### A “culture of criticism”

Smith (2006) described peer review as “a flawed process, full of easily identified defects with little evidence that it works” (p. 182). Yet peer review provides one way of forcing researchers to recognise when they are so focused on a favoured theory that they are unable to break away. Hossenfelder (2018) has argued that the field of particle physics has stagnated because of a reluctance to abandon theories that are deemed “beautiful.” We are accustomed to regarding physicists as superior to psychologists in terms of theoretical and methodological sophistication. In general, they place far less emphasis than we do on statistical criteria for evidence, and where they do use statistics, they understand probability theory and adopt very stringent levels of significance. Nevertheless, according to Hossenfelder, they are subject to cognitive and social biases that make them reluctant to discard theories. She concludes her book with an Appendix on “What you can do to help,” and as well as advocating better understanding of cognitive biases, she recommends some cultural changes to address these. These include building “a culture of criticism.” In principle, we already have this—talks and seminars should provide a forum for research to be challenged—but in practice, critiquing another’s work is often seen as clashing with social conventions of being supportive to others, especially when it is conducted in public.

Recently, two other approaches have been developed, with the potential to make a “culture of criticism” more useful and more socially acceptable. Registered Reports (Chambers, 2019) is an approach that was devised to prevent publication bias, *p*-hacking, and HARKing. This format moves the peer review process to a point before data collection so that results cannot influence editorial decisions. An unexpected positive consequence is that peer review comes at a point when it can be acted upon to improve the experimental design. Where reviewers of Registered Reports ask “how could we disprove the hypothesis?” and “what other explanations should we consider?” this can generate more informative experiments.

A related idea is borrowed from business practices and is known as the “pre mortem” approach (Klein, 2007). Project developers gather together and are asked to imagine that a proposed project has gone ahead and failed. They are then encouraged to write down reasons why this has happened, allowing people to voice misgivings that they may have been reluctant to state openly, so they can be addressed before the project has begun. It would be worth evaluating the effectiveness of pre-mortems for scientific projects. We could strengthen this approach by incorporating ideas from Bang and Frith (2017), who noted that group decision-making is most likely to be effective when the group is diverse and people can express their views anonymously. With both Registered Reports and the study pre-mortem, reviewers can have a role as critical friends who can encourage researchers to identify ways to improve a project before it is conducted. This can be a more positive experience for the reviewer, who may otherwise have no option but to recommend rejection of a study with flawed methodology.

### Counteracting cherry-picking of literature

Turning to cherry-picking of prior literature, the established solution is the systematic review, where clear criteria are laid out in advance so that a comprehensive search can be made of all relevant studies (Siddaway, Wood, & Hedges, 2019). The systematic review is only as good as the data that go into it, however, and if a field suffers from substantial publication bias and/or *p*-hacking, then, rather than tackling error entrenchment, it may add to it. With the most scrupulous search strategy, relevant papers with null results can be missed because positive results are mentioned in titles and abstracts of papers, whereas null results are not (Lazic, 2016, p. 15). This can mean that, if a study is looking at many possible associations (e.g., with brain regions or with genes), studies that considered a specific association but failed to find support for it will be systematically disregarded. This may explain why it seems to take 30 or 40 years for some erroneous entrenched theories to be abandoned. The situation may improve with increasing availability of open data. Provided data are adequately documented and accessible, the problem of missing relevant studies may be reduced.

Ultimately, the problem of biased reviews may not be soluble just by changing people’s citation habits. Journal editors and reviewers could insist that abstracts follow a structured format and report all variables that were tested, not just those that gave significant results. A more radical approach by funders may be needed to disrupt this wasteful cycle. When a research team applies to test a new idea, they could first be required to (a) conduct a systematic review (unless one has been recently done) and (b) replicate the original findings on which the work is based: this is the opposite to what happens currently, where novelty and originality are major criteria for funding. In addition, it could be made mandatory for any newly funded research idea to be investigated by at least two independent laboratories and using at least two different approaches (triangulation). All these measures would drastically slow down science and may be unfeasible where research needs highly specialised equipment, facilities, or skills that are specific to one laboratory. Nevertheless, slower science may be preferable to the current system where there are so many examples of false leads being pursued for decades, with consequent waste of resources.

### Reconciling storytelling with honesty

Perhaps the hardest problem is how to reconcile our need for narrative with a “warts and all” account of research. Consider this advice from Bem (2004)—which I suspect many journal editors would endorse:

Contrary to the conventional wisdom, science does not care how clever or clairvoyant you were at guessing your results ahead of time. Scientific integrity does not require you to lead your readers through all your wrongheaded hunches only to show—voila!—they were wrongheaded. A journal article should not be a personal history of your stillborn thoughts . . .

Your overriding purpose is to tell the world what you have learned from your study. If your results suggest a compelling framework for their presentation, adopt it and make the most instructive findings your centerpiece . . . Think of your dataset as a jewel. Your task is to cut and polish it, to select the facets to highlight, and to craft the best setting for it.

As Kerr (1998) pointed out, HARKing gives a misleading impression of what was found, which can be particularly damaging for students, who on reading literature may form the impression that it is normal for scientists to have their predictions confirmed and think of themselves as incompetent when their own experiments do not work out that way. One of the goals of pre-registration is to ensure that researchers do not omit inconvenient facts when writing up a study—or if they do, at least make it possible to see that this has been done. In the field of clinical medicine, impressive progress has been made in methodology, with registration now a requirement for clinical trials (International Committee of Medical Journal Editors, 2019). Yet, Goldacre et al. (2019) found that even when a trial was registered, it was common for researchers to change the primary outcome measure without explanation, and it has been similarly noted that pre-registrations in psychology are often too ambiguous to preclude *p*-hacking (Veldkamp et al., 2018). Registered Reports (Chambers, 2019) adopt stricter standards that should prevent HARKing, but the author may struggle to maintain a strong narrative because messy reality makes a less compelling story than a set of results subjected to Bem’s (2004) cutting and polishing process.

### Rewarding credible research practices

A final set of recommendations has to do with changing the culture so that incentives are aligned with efforts to counteract unhelpful cognitive constraints, and researchers are rewarded for doing reproducible, replicable research, rather than for grant income or publications in high-impact journals (Forstmeier, Wagenmakers, & Parker, 2016; Pulverer, 2015). There is already evidence that funders are concerned to address problems with credibility of biomedical research (Academy of Medical Sciences, 2015), and rigour and reproducibility are increasingly mentioned in grant guidelines (e.g., https://grants.nih.gov/policy/reproducibility/index.htm). One funder, Cancer Research UK, is innovating by incorporating Registered Reports in a two-stage funding model (Munafò, 2017). We now need publishers and institutions to follow suit and ensure that researchers are not disadvantaged by adopting a self-critical mind-set and engaging in practices of open and reproducible science (Poldrack, 2019).
